# Effect of nurse-led telephone follow-up on postoperative symptoms and analgesics consumption after benign hysterectomy: a randomized, single-blinded, four-arm, controlled multicenter trial

**DOI:** 10.1007/s00404-022-06722-x

**Published:** 2022-09-02

**Authors:** Gulnara Kassymova, Gunilla Sydsjö, Ninnie Borendal Wodlin, Lena Nilsson, Preben Kjølhede

**Affiliations:** 1 Department of Obstetrics and Gynecology in Linköping, Department of Biomedical and Clinical Sciences, Linköping University, University Hospital, S-58245 Linköping, Sweden; 2 Department of Anesthesiology and Intensive Care in Linköping and Department of Biomedical and Clinical Sciences, Linköping University, Linköping, Sweden

**Keywords:** Coaching, ERAS, Hysterectomy, Postoperative symptoms, Telephone follow-up

## Abstract

**Purpose:**

The study aimed to determine if planned telephone follow-up, especially when adding structured, oriented coaching, reduces the intensity of postoperative symptoms and decreases analgesics consumption after benign hysterectomy.

**Methods:**

A randomized, single-blinded, four-armed, controlled multicenter trial of 525 women scheduled for hysterectomy was conducted in 5 hospitals in the southeast health region of Sweden. The women were allocated 1:1:1:1 into four follow-up models: (A) no telephone follow-up (control group); (B) one planned, structured, telephone follow-up the day after discharge; (C) as B but with additional telephone follow-up once weekly for 6 weeks; and (D) as C but with oriented coaching telephone follow-up on all occasions. Postoperative symptoms were assessed using the Swedish Postoperative Symptoms Questionnaire. Analgesic consumption was registered. Unplanned telephone contacts and visits were registered during the 6 weeks of follow-up.

**Results:**

In total, 487 women completed the study. Neither pain intensity, nor symptom sum score or analgesic consumption differed between the intervention groups. Altogether, 224 (46.0%) women had unplanned telephone contacts and 203 (41.7%) had unplanned visits. Independent of intervention, the women with unplanned telephone contacts had higher pain intensity and symptom sum scores, particularly if an unplanned telephone contact was followed by a visit, or an unplanned visit was preceded by an unplanned telephone contact.

**Conclusion:**

Telephone follow-up did not seem to affect recovery regarding symptoms or analgesic consumption after benign hysterectomy in an enhanced recovery after surgery (ERAS) setting. Unplanned telephone contacts and visits were associated with more postoperative symptoms, especially pain.

*Trial registration* The study is registered in ClinicalTrial.gov: NCT01526668 retrospectively from January 27; 2012. Date of enrolment of first patient: October 11; 2011.

## What does this study add to the clinical work

**Table:** 

Nurse-led telephone follow-up has no impact on the trajectory of postoperative symptoms and analgesic consumption after benign hysterectomy. Unplanned telephone contacts and visits to health care providers postoperatively are associated with postoperative symptoms, in particular pain.	

## Introduction

Patients may still experience discomfort in the recovery period after hysterectomy in spite of the use of enhanced recovery after surgery (ERAS) multimodal programs aimed at minimizing the pathophysiological changes associated with surgery [1]. A variety of symptoms, e.g. pain, postoperative nausea and vomiting, fatigue, gastrointestinal symptoms, and itching, are commonly reported after hysterectomy [2]. Thus, it seems necessary to add interventions that may influence the experience of postoperative symptoms.

The early and intermediate phases of recovery take place while the patient is still in hospital [3, 4]. In the late recovery phase, i.e., after discharge from hospital, the patient self-manages the postoperative symptoms using the information/education obtained through the ERAS program and the prescribed medication. If the provided treatment is inadequate or new discomforts occur, the patient may initiate contact with a health care provider.

Routine follow-up contacts after hysterectomy on benign indication are not common praxis in Sweden. However, qualitative studies have indicated that women strongly believe that the recovery after surgery would benefit from follow-up contact by nurses [5–8]. Gynecological clinics may, therefore, be attempted to implement nurse-led telephone follow-up (TFU) after discharge. The content of TFU has not been standardized or evaluated, and no evidence for the benefit of such contacts has been reported. We performed a randomized controlled four-armed multicenter trial (the Post-hysterectomy recovery (POSTHYSTREC) trial), comparing three nurse-led TFU programs and a control with no TFU after benign hysterectomy, to investigate whether TFU in an ERAS setting accelerates recovery. No effect on recovery of health-related quality of life of the four modes of follow-up was found, but the number of women with unplanned telephone contacts after discharge (uTC) was significantly lower after a structured oriented coaching TFU [9]. The present study examines secondary outcomes from the POSTHYSTREC trial.

The primary aim of this study was to determine if different nurse-led TFU strategies, including structured oriented coaching, affect postoperative symptoms. The secondary aims were to determine the impact of TFUs on consumption of analgesics, and to analyze the impact on postoperative symptoms and consumption of analgesics in women who had uTC and unplanned visits (uV) within 6 weeks after discharge from the hospital.

## Materials and methods

The POSTHYSTREC trial, a randomized single-blinded, controlled multicenter intervention trial of women undergoing hysterectomy on benign indication, was undertaken at the departments of obstetrics and gynecology in five hospitals in the southeast region of Sweden between October 2011 and May 2017. The Regional Ethics Board of Linköping University approved the study (Dnr.2011/106-31), and the study was registered in ClinicalTrial.gov: (NCT01526668).

The study has previously been described in detail [9]. Briefly, inclusion criteria were women scheduled for abdominal (total or subtotal), or vaginal hysterectomy, aged 18 to 60 years, receiving written and verbal information and providing signed informed consent. Speaking Swedish fluently and having access to a phone or the internet were requirements. Exclusion criteria were women scheduled for hysterectomy due to genital prolapse, cancer heredity, or suspected/confirmed invasive gynecological malignancies, previous or planned bilateral oophorectomy, mental and physical disability, severe mental illness, and current drug or alcohol abuse.

The pre- and postoperative care followed the ERAS program [10, 11]. The mode of hysterectomy was decided by the gynecologist after consultation with the patient before enrolment in the study.

A computer generated the randomization code [12] with an allocation ratio of 1:1:1:1. The allocated intervention was written on a paper enclosed in consecutively numbered sealed opaque envelopes. A block randomization method was used, with allocation and stratification for center and abdominal or vaginal hysterectomy. The participants were randomized before surgery in order of the numbered envelopes. All participants were informed that there would be follow-up contact with the research nurse (RN) postoperatively but the frequency of the follow-up contact was concealed, and was first revealed to the woman at the time of opening the randomization envelope at discharge. Thus, the participants were blinded to the content and the frequency of the follow-up contact.

Women were randomized to one of four follow-up programs.

- Group A—no planned follow-up contact after discharge. The patient was requested to initiate contact if necessary.
- Group B—one planned TFU with the RN the day after discharge. Thereafter, the patient was requested to initiate contact if necessary.
- Group C—planned TFU with the RN the day after discharge and then once weekly for 6 weeks.
- Group D—planned, structured, oriented coaching TFU with the RN the day after discharge, and then once weekly for 6 weeks.

The content of the TFU and coaching, including education of the RNs, is described in detail [9]. The structured, oriented coaching was an experimental intervention based on elements from Cognitive Behavior Therapy (CBT) and clinical experience.

All women were seen by the RN for collection of the questionnaires and the diary at the end of the trial 6 weeks postoperatively.

### Swedish postoperative symptoms questionnaire (SPSQ)

The SPSQ is a validated questionnaire that measures common postoperative symptoms such as nausea, retching, headache, abdominal pain, tiredness, drowsiness, blurred vision, and itching [1, 2]. The intensity of each symptom was rated as ‘none’ (1), ‘yes, a little’ (2), ‘yes, somewhat’ (3) and ‘yes, a ‘lot’ (4). The sum score of these eight symptoms, which constituted a measurement of the overall discomfort, ranged between 8 and 32: the higher the sum score, the greater the discomfort. The women reported the maximum experienced intensity of the pain, i.e., when the pain was at its worst, and how the pain was felt on average on the particular day. The maximum and average pain intensities were rated on a seven-point scale as: “none” (0), “very mild” (1), “mild” (2), “moderate” (3), “bad” (4), “severe” (5), and “very severe” (6). The SPSQ questionnaire was completed once daily at the same time during the first postoperative week, starting the evening after surgery and thereafter once weekly until 6 weeks postoperatively.

The women received standardized postoperative analgesics consisting of oral paracetamol, 665 mg, two tablets three times daily, diclofenac, 50 mg, three times daily and oxycodone, 10–20 mg, twice daily. At discharge, the patient received prescriptions of oral non-opioids (paracetamol and diclofenac) and if necessary weak opioid-containing analgesics (codeine or tramadol). Consumption of analgesics was registered in the patient’s record during hospitalization. After discharge, the patient reported the daily consumption in a diary. The daily dose of opioids was converted into an equivalent intravenous dose of morphine and the daily dose of non-opioids was converted to the World Health Organization’s defined daily dose [13].

Demographic and clinical data were collected prospectively from the time of inclusion until the scheduled end-of-study visit 6 weeks after the hysterectomy. uTCs and uVs were registered, defined as any emergency or non-pre-arranged telephone contact or visit due to a medical reason with a health care provider in inpatient or outpatient care or primary care (doctors, nurses, or assistant nurses) after discharge up to the 6-week follow-up visit.

### Outcome measures

Primary outcome measures were maximum and average pain intensities and symptom sum score. Secondary outcomes were consumption of opioid and non-opioid analgesics, uTCs, and uVs.

### Statistical analyses

Data were analyzed using the software TIBCO Statistica® 13.5.0 (TIBCO Software Inc., Palo Alto, CA 94,304 USA). Measures of central tendency and dispersion are reported as mean and standard deviation. Categorical data are presented as number and percent. Comparison of groups was done by use of one-way analysis of variance (ANOVA) for normal distributed continuous data and Kruskal–Wallis ANOVA for non-normally distributed continuous data. Nominal data were analyzed by means of Pearson’s chi-squared tests.

Data measured on repeated occasions were analyzed by means of repeated measures ANOVA. Post hoc tests for between-group comparisons were conducted using Tukey’s honestly significant difference tests. Non-normally distributed variables were logarithmically transformed and used as such in the analysis. The repeated measures ANOVA models were adjusted for mental disorder, mode of surgery, consumption of opioids on day 2–7, non-opioids on days 2–15, day of discharge (categorized), and intervention. The level of significance was set at *p* < 0.05 for two-tailed tests.

Missing data of an item in the SPSQ on an occasion of measurement were replaced by the mean value of the item of the intervention group on the specific occasion. The number of missing cells in the SPSQ made up 3.2% concerning maximum pain intensity, 3.5% for average pain intensity, and 4.1% for the items of the symptom sum score.

### Power analysis

Sample size estimation of the POSTHYSTREC trial was based on the primary outcomes of the trial (quality of life measures and duration of sick leave) and described in detail [9]. No power analyses were conducted for the outcome measures of the present study.

## Results

**Fig. 1:**
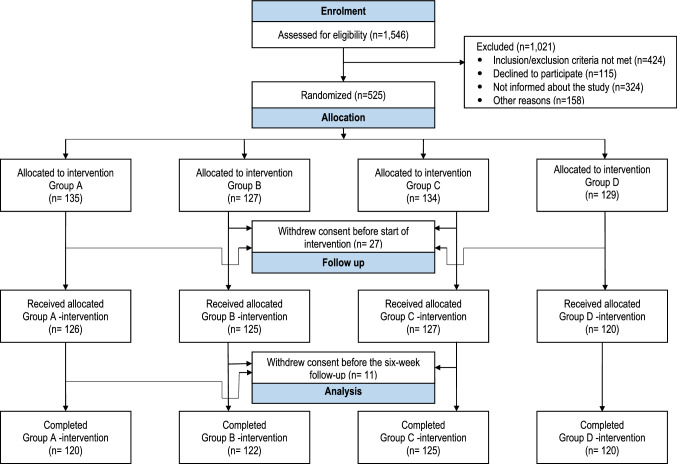
CONSORT flowchart of participants in the POSTHYSTREC trial

The flowchart of the study population is presented in Fig. 1. Demographic and clinical data are reported in Table 1. The groups were balanced in baseline data except for the preoperative occurrence of mental disorder in 19.2%, 6.6%, 16.0% and 11.7% of Groups A, B, C and D, respectively. The daily consumption of analgesics postoperatively and background data concerning outcomes of the SPSQ on day 0 (day of surgery) and day 1 are presented in Table 2. Opioid consumption is shown for only 8 days and the non-opioid consumption for 16 days due to very low consumption of the respective analgesics after these days.

**Table 1:** Demographic and clinical descriptive data of 487 women undergoing benign hysterectomy subdivided after intervention group

		Group A (*n* = 120)	Group B (*n* = 122)	Group C (*n* = 125)	Group D (*n* = 120)
Age (years)		45.5 (5.3)	47.2 (5.6)	46.2 (5.3)	47.0 (5.8)
< 40 years		18 (15.0)	14 (11.5)	21 (16.8)	14 (11.7)
Between 40 and 50 years		83 (59.2)	76 (62.3)	79 (63.2)	76 (63.3)
> 50 years		19 (15.8)	32 (26.2)	25 (20.0)	30 (25.0)
BMI (kg/m^2^)		26.8 (4.8)	27.0 (4.8)	26.7 (4.6)	26.5 (4.6)
≤ 25.0 kg/m^2^		53 (44.1)	49 (40.2)	60 (48.0)	56 (46.7)
> 25.0 and – 29.9 kg/m^2^		38 (31.7)	43 (35.2)	35 (28.0)	40 (33.3)
≥ 30.0 kg/m^2^		29 (24.2)	30 (24.6)	30 (24.0)	24 (20.0)
Nulliparous		10 (8.4)	19 (15.8)	15 (12.0)	20 (16.7)
Smoking		18 (15.5)	9 (7.6)	18 (14.4)	11 (9.6)
Gainfully employment	Yes	107 (89.2)	117 (95.9)	111 (88.8)	113 (94.2)
	No	13 (10.8)	5 (4.1)	14 (11.2)	7 (5.8)
Co-morbidity	Mental disorders†	23 (19.2)	8 (6.6)	20 (16.0)	14 (11.7)
	Chronic pain disorder	28 (23.3)	30 (24.6)	29 (23.2)	31 (25.8)
Taking medicine regularly preoperatively					
Analgesics	Non-opioid analgesics	14 (11.7)	20 (16.4)	22 (17.6)	11 (9.2)
	Opioid analgesics	4 (3.3)	3 (2.5)	6 (4.8)	6 (5.0)
Hypnotics		8 (6.7)	2 (1.6)	7 (5.6)	7 (5.8)
Previous laparotomy		39 (32.8)	37 (30.5)	46 (37.4)	39 (32.8)
Hysterectomy indication	Myoma uteri	58 (48.3)	65 (53.3)	47 (37.6)	53 (44.2)
Bleeding disorder		32 (26.7)	23 (18.8)	35 (28.0)	35 (29.2)
Myoma and bleeding		10 (8.3)	14 (11.5)	21 (16.8)	13 (10.8)
Cervical dysplasia		14 (11.7)	12 (9.8)	14 (11.2)	9 (7.5)
Pain		5 (4.2)	8 (6.6)	8 (6.4)	9 (7.5)
Others		1 (0.8)	0 (0.0)	0 (0.0)	1 (0.8)
ASA classification	Class 1	84 (70.0)	78 (63.9)	79 (63.2)	79 (65.8)
	Class 2	35 (29.2)	40 (32.8)	39 (31.2)	39 (32.5)
	Class 3	1 (0.8)	4 (3.3)	7 (5.6)	2 (1.7)
Mode of hysterectomy	Abdominal	97 (80.8)	98 (80.3)	98 (78.4)	90 (75.0)
	Vaginal	23 (19.2)	24 (19.7)	27 (21.6)	30 (25.0)
Mode of anesthesia	GA	55 (45.8)	37 (30.3)	50 (40.0)	41 (34.2)
	ITA + IT morphine	34 (28.4)	52 (42.6)	45 (36.0)	49 (40.8)
	GA + IT morphine	31 (25.8)	33 (27.1)	30 (24.0)	30 (25.0)
Hospital stay (days)		1.8 (1.1)	1.8 (1.3)	1.6 (1.0)	1.6 (1.2)
Day of discharge*	Day 1	47 (39.2)	53 (43.4)	50 (40.0)	64 (53.3)
	Day 2	60 (50.0)	55 (45.1)	62 (49.6)	48 (40.0)
	Day 3	8 (6.7)	10 (8.2)	7 (5.6)	7 (5.8)
	Day 4 or later	5 (4.2)	4 (3.3)	6 (4.8)	1 (0.8)
Intraoperative complications#		7 (5.8)	8 (6.6)	3 (2.4)	3 (2.5)
Postoperative complications (Clavien-Dindo)^‡^	I	17 (14.2)	15 (12.3)	16 (12.8)	9 (7.5)
	II	17 (14.2)	19 (15.6)	24 (19.2)	16 (13.3)
	III	3 (2.5)	6 (4.9)	4 (3.2)	3 (2.5)

Figures denote mean and (standard deviation) or number of women and (percent)

*ASA* American Society of Anesthesiologists, *GA* general anesthesia, *IT*, intrathecal, *ITA* intrathecal anesthesia

^#^Intraoperative complications include intraoperative bleeding exceeding 1000 mL, large vessel or organ damage

^†^Mental disorders comprise minor mental disorders such as anxiety, panic, depressive and mood disorders, and phobias

*Day of discharge: Day of surgery = Day 0

^‡^Clavien-Dindo contracted classification of postoperative complications

**Table 2:** (A) Daily consumption of opioids (equivalent iv. morphine dose (mg)) and non-opioids (in DDD) day-by day in 487 women undergoing benign hysterectomy in relation to intervention group, and (B) background data concerning outcomes of the SPSQ on day 0 (day of surgery) and day 1

	Day^†^	Group A (*n* = 120)	Group B (*n* = 122)	Group C (*n* = 125)	Group D (*n* = 120)	*p*-value*
(**A**)						
Equivalent iv. morphine (mg)	0	10.1 (12.2)	7.7 (11.3)	8.6 (10.0)	8.9 (13.0)	0.41
	1	4.7 (7.4)	4.5 (7.2)	5.8 (8.6)	4.1 (6.0)	0.13
	2	2.9 (5.8)	3.3 (7.4)	3.8 (6.3)	2.8 (5.6)	0.17
	3	2.1 (4.7)	1.8 (4.6)	2.2 (4.6)	2.2 (4.8)	0.42
	4	1.4 (3.8)	1.8 (6.5)	1.6 (4.3)	1.5 (3.7)	0.61
	5	0.8 (2.7)	1.3 (5.3)	1.0 (3.0)	0.9 (3.6)	0.44
	6	0.7 (2.6)	0.9 (4.9)	0.7 (2.6)	0.2 (1.1)	0.21
	7	0.4 (1.7)	0.9 (4.6)	0.7 (2.6)	0.4 (2.5)	0.32
Non-opioids (DDD)	0	1.7 (0.9)	1.9 (0.8)	1.7 (1.1)	1.8 (0.9)	0.21
	1	2.3 (0.9)	2.1 (1.0)	2.1 (1.0)	2.2 (1.0)	0.32
	2	2.1 (1.0)	2.2 (1.1)	2.1 (1.1)	2.1 (1.2)	0.77
	3	1.9 (1.1)	1.9 (1.1)	2.0 (1.1)	2.0 (1.1)	0.93
	4	1.9 (1.1)	1.7 (1.1)	1.8 (1.1)	1.9 (1.1)	0.68
	5	1.7 (1.1)	1.6 (1.2)	1.6 (1.1)	1.7 (1.1)	0.56
	6	1.5 (1.1)	1.4 (1.1)	1.5 (1.1)	1.4 (1.2)	0.88
	7	1.4 (1.1)	1.4 (1.1)	1.4 (1.1)	1.2 (1.1)	0.35
	8	1.2 (1.1)	1.2 (1.0)	1.2 (1.1)	1.1 (1.1)	0.98
	9	1.1 (1.0)	1.1 (1.0)	1.0 (1.0)	1.0 (1.1)	0.65
	10	0.9 (0.9)	1.0 (1.0)	0.9 (0.9)	0.9 (1.0)	0.64
	11	0.8 (0.9)	0.9 (0.9)	0.8 (0.9)	0.8 (1.0)	0.66
	12	0.7 (0.8)	0.8 (0.9)	0.8 (0.9)	0.7 (0.9)	0.78
	13	0.5 (0.8)	0.7 (0.9)	0.6 (0.9)	0.6 (0.8)	0.64
	14	0.5 (0.7)	0.6 (0.8)	0.6 (0.8)	0.5 (0.8)	0.80
	15	0.4 (0.7)	0.4 (0.7)	0.5 (0.8)	0.4 (0.8)	0.90
(**B**)						
Maximum pain intensity	0	3.8 (1.6)	3.0 (1.7)	3.4 (1.7)	3.2 (1.6)	< 0.001
	1	3.4 (1.5)	3.4 (1.5)	3.3 (1.5)	3.3 (1.2)	0.90
On average pain intensity	0	3.0 (1.4)	2.6 (1.7)	2.8 (1.5)	2.6 (1.4)	0.11
	1	2.9 (1.3)	2.8 (1.3)	2.7 (1.3)	2.8 (1.2)	0.82
Symptom sum score	0	16.5 (4.7)	15.5 (4.5)	15.9 (4.1)	16.2 (3.6)	0.26
	1	14.2 (4.2)	13.8 (4.0)	14.1 (4.0)	13.7 (3.6)	0.76

Figures denote mean and (standard deviation)

*DDD* defined daily dose

^†^Day 0 = day of surgery

*Kruskal Wallis ANOVA

**Fig. 2:**
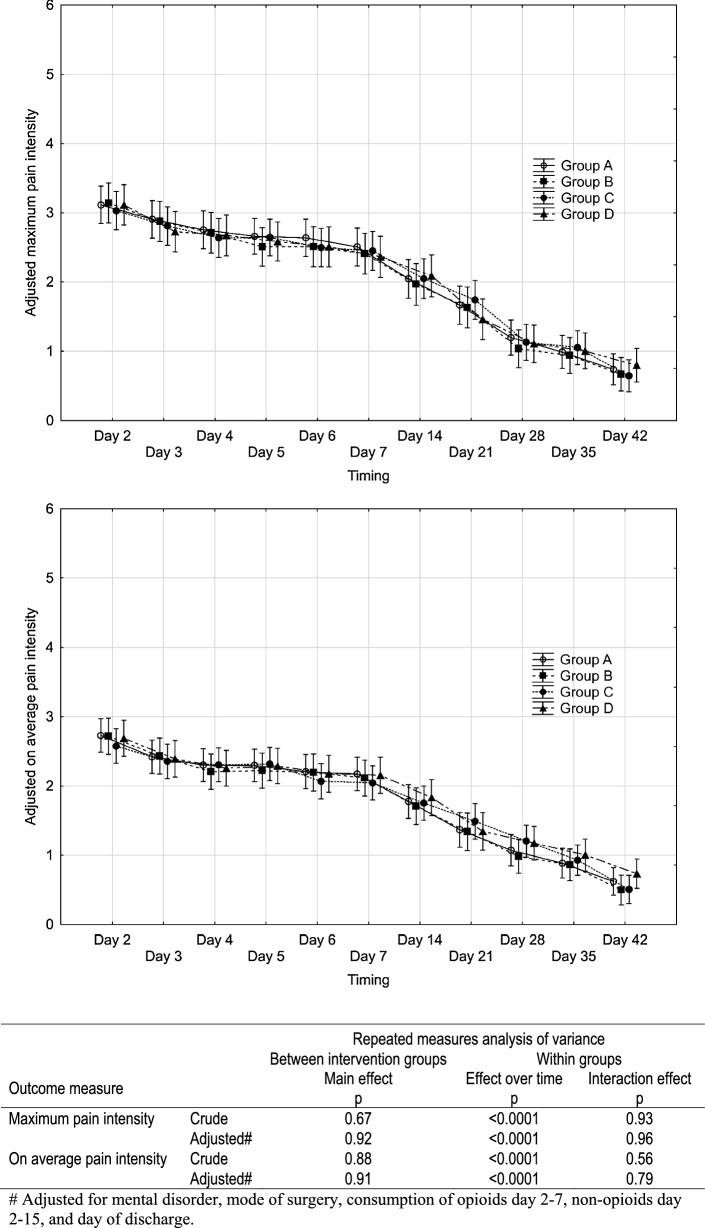
Maximum pain intensity and average pain intensity after benign hysterectomy from day 2 to day 42 postoperatively in relation to invention group. Pain intensity measured on a scale from 0 (no pain) to 6 (very severe pain). Results of repeated measures ANOVA are shown in the table below the diagrams

**Fig. 3:**
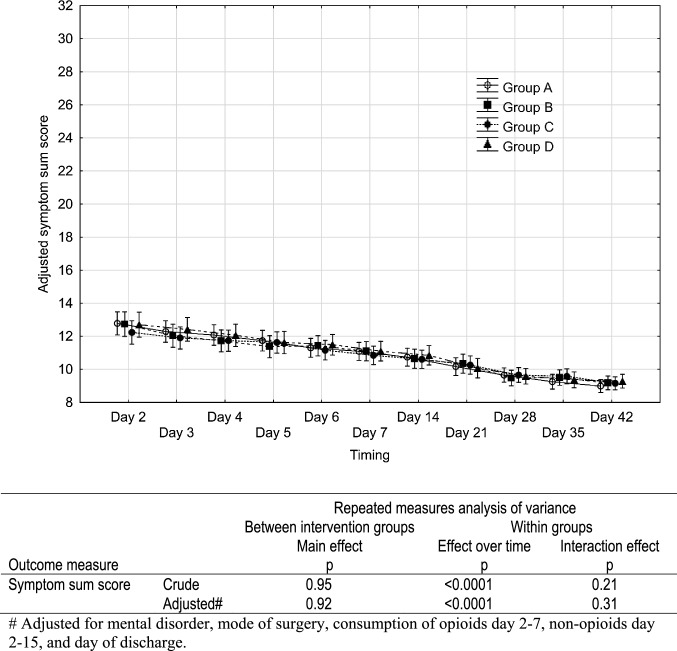
Symptom sum score postoperatively after benign hysterectomy in relation to intervention group. Symptom sum score ranges from 8 to 32. The higher the sum score the more symptoms. Results of repeated measures ANOVA are shown in the table below the diagram

**Fig. 4:**
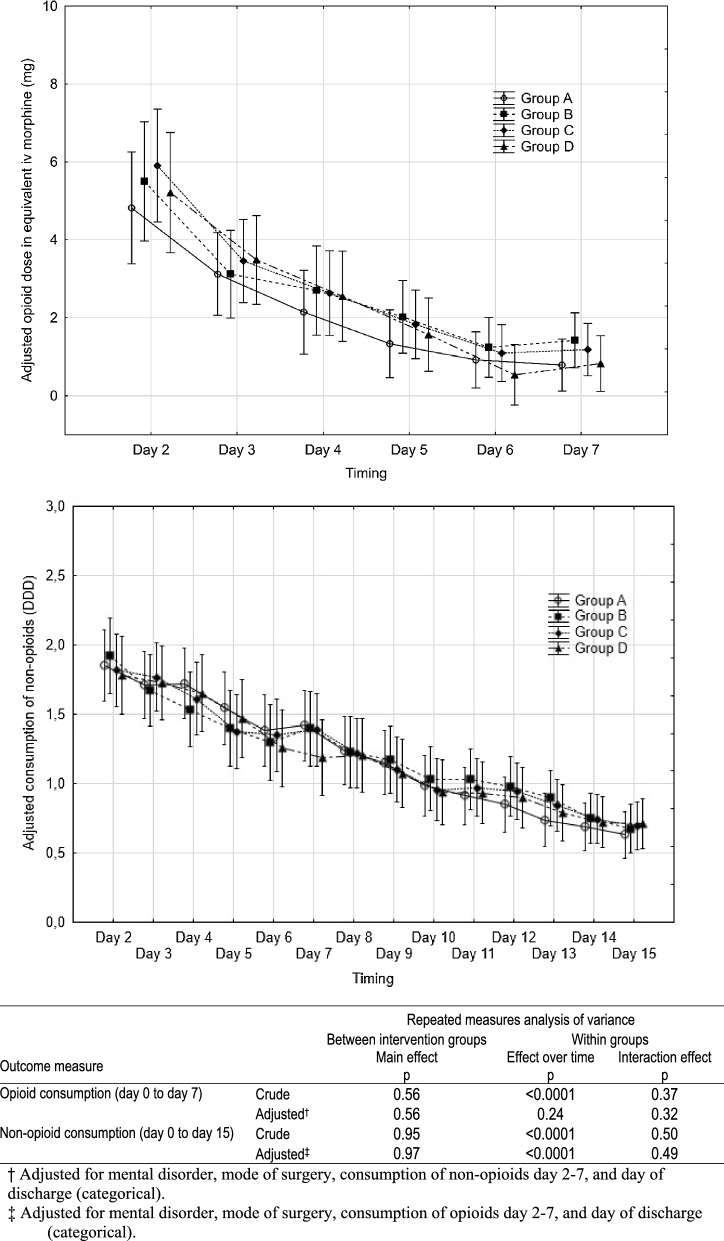
Consumption of opioids (in equivalent intravenous morphine (mg)) and non-opioids (in defined daily dose according to WHO [13]) after benign hysterectomy in relation to intervention group. Results of repeated measures ANOVA are shown in the table below the diagrams

No significant difference was found between the intervention groups in any of the recovery outcomes (maximum pain intensity, average pain intensity, symptom sum score, and consumption of opioids and non-opioids), as shown in Figs. 2, 3, 4. The results remained when adjusted for mental disorder, mode of hysterectomy, consumption of opioids and non-opioids, and day of discharge. The level of the outcome measures decreased significantly over time, and no interaction effects were observed.

By adding the other factors depicted in Table 1 separately into the adjusted repeated measures ANOVA models, the outcome of the intervention remained insignificant, indicating that no subgroup among the factors would benefit from the intervention (data not shown).

The occurrence of uTC and uV is presented in Table 3A. In total, 224 (46.0%) women had a uTC and 203 (41.7%) women had a uV.

**Table 3:** (A) Occurrence of unplanned telephone contact (uTC) and unplanned visit (uV), categorized into 3 groups according to following visit and preceded telephone contact, respectively, in 487 women undergoing hysterectomy. (B) presents the outcome of repeated measures analysis of variance for the outcome measures in relation to uTC groups, and (C) the corresponding for uV

A	No uTC	uTC without a following visit	uTC with a following visit
uTC	263 (54.0%)	80 (16.4%)	144 (29.6%)
	No uV	uV without a preceded telephone contact	uV with a preceded telephone contact
uV	284 (58.3%)	59 (12.1%)	144 (29.6%)

B. Unplanned telephone contact	Repeated measures analysis of variance			
	Between the three uTC groups	Within groups		Effect of intervention
	Main effect	Effect over time	Interaction effect	
Outcome measure	*p*-value	*p*-value	*p*-value	*p*-value
Maximum pain intensity#	< 0.0001	< 0.0001	0.51	0.97
On average pain intensity#	< 0.0001	< 0.0001	0.85	0.80
Symptom sum score#	< 0.0001	< 0.0001	0.25	0.27
Opioid consumption^†^	0.30	0.21	0.79	0.56
Non-opioid consumption^‡^	0.81	< 0.0001	< 0.01	0.96

C. Unplanned visit	Repeated measures analysis of variance			
	Between the three uV groups	Within groups		Effect of intervention
	Main effect	Effect over time	Interaction effect	
Outcome measure	*p*-value	*p*-value	*p*-value	*p*-value
Maximum pain intensity#	< 0.0001	< 0.001	0.36	0.90
On average pain intensity#	< 0.0001	< 0.001	0.72	0.81
Symptom sum score#	< 0.0001	< 0.0001	0.40	0.75
Opioid consumption^†^	0.18	0.19	0.76	0.58
Non-opioid consumption^‡^	0.91	< 0.0001	0.08	0.94

^#^Measured daily from day 2 after surgery to day 7 and then once weekly until 6 weeks postoperatively. Adjusted for mental disorder, mode of surgery, consumption of opioids day 2–7, non-opioids day 2–15, day of discharge (categorical), and intervention

^†^Opioid consumption day 2 to day 7, adjusted for mental disorder, consumption of non-opioids day 2–7, day of discharge (categorical), and intervention

^‡^Non-opioid consumption day 2 to day 15, adjusted for mental disorder, consumption of opioids day 2-7, day of discharge (categorical), and intervention

The results of the repeated measures ANOVA of the recovery outcome measures in relation to the grouping of uTCs or uVs and the influence of the interventions are presented in Tables 3B, C. Significant associations were found between the groups concerning maximum and average pain intensity and symptom sum scores for both uTCs and uVs, whereas consumption of opioids and non-opioids did not differ between groups. The interventions per se did not independently affect any of the recovery outcome measures in the uTC or the uV groups.

**Fig. 5:**
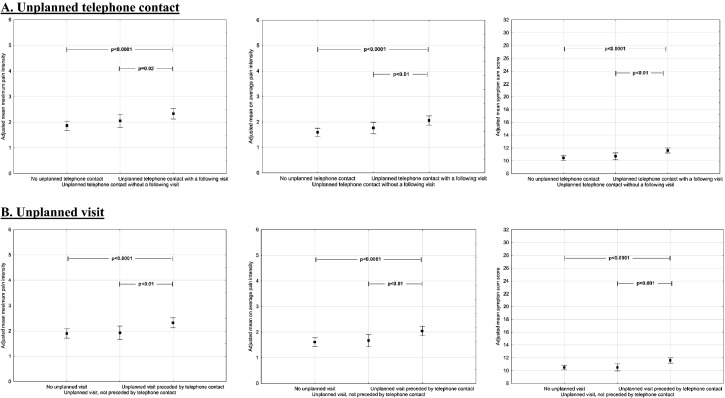
Post hoc tests of the significant outcomes depicted in Table 3 between groups, (**A**) with and without unplanned telephone contacts, and (**B**) with and without unplanned visits. Plots indicate means and bars represent 95% confidence interval

The results of the post hoc analyses are graphically illustrated in Fig. 5. In summary, the maximum and average pain intensity, and the symptom sum score were significantly higher in the women who had a uTC followed by a visit than in both the women who had no uTC (*p* < 0.0001 for all) and those with a uTC without a following visit (*p* = 0.02 and *p* < 0.01 for the pain intensities, and symptom sum score). Similar findings were seen in the women who had a uV preceded by a telephone contact versus the women who had no uV (*p *< 0.0001 for all) and those with a uV without a preceding telephone contact (*p* < 0.01 for both pain intensities, and *p* < 0.001 for symptom sum score). By contrast, no differences were seen between those with no uTC and those with a uTC without a following visit, and between those with no uV and those with a uV without a preceding telephone contact, respectively.

## Discussion

This study showed that interventions with a planned nurse-led TFU including oriented structured coaching after benign hysterectomy did not decrease the level of intensity of postoperative pain, the summary score of common postoperative symptoms or the consumption of analgesics, more than in women who had no planned TFU. Women who had a uTC followed by a visit to a health care provider within 6 weeks of surgery had significantly higher levels of pain intensity and a higher summary score of postoperative symptoms, but showed no difference in analgesics consumption, compared to the women who had no uTC or those with a uTC without a following visit. These results were not influenced by the interventions in spite of a significantly lower occurrence of uTCs in women who had a planned TFU, in particular when using structured oriented coaching TFU [9]. Similar results were seen concerning postoperative uVs in health care facilities.

The use of a randomized controlled design, the large number of patients, and the use of repeated measurements constitute major strengths of the study. Moreover, the trial was intended, in an innovative way, to adapt clinical use of an experimental treatment concept, founded on evidence-based methods. The lack of an a priori power calculation to estimate sample size for the objectives of this study might be seen as a drawback. However, the differences in outcomes between the intervention groups were small and lacked clinical significance. This might support our interpretation of the results.

The content of the TFU may be seen as a limitation, because it has not been validated. However, the content of the standard TFU equals what is used by the nurses in daily clinical practice, whereas the structured oriented coaching TFU is experimental. The structured oriented coaching TFU, which was based on elements derived from CBT, was developed by a clinically and scientifically experienced CBT therapist (GS). We, therefore, believe that both methods had a firm clinical and theoretical foundation and the structured oriented coaching TFU theoretically should be effective in improving recovery.

The single blinding design may be seen as a strength but also a limitation. There is also a risk that the RNs may have mixed up the content of the structured oriented coaching TFU with the standard follow-up strategy components. To prevent this, GS had several education sessions with the RNs where the importance of avoiding a mix of the strategies was emphasized.

Previous research on TFU has evaluated a variety of outcomes for TFU, including enhancing patient safety, improving the quality and continuity of care, reducing adverse events, clarifying patient understanding of discharge care instructions, seeking patient feedback about their experience of care, and addressing any concerns about their experience or their recovery [14]. Three systematic reviews have been published concerning TFU after surgery and discharge from hospital [15–17]. The systematic reviews drew similar conclusions that the methodological quality of the included studies was low, they lacked valid and reliable tools to assess patient outcomes, and there was limited evidence of the benefits of TFU.

To the best of our knowledge, no previous study has evaluated the effect of TFU on postoperative recovery from symptoms and analgesic consumption after hysterectomy. However, we did not observe any benefit of TFU on recovery from postoperative pain, and the summary score of symptoms or analgesics consumption at any time, nor did we find any subgroup that would benefit. Although these results indicate that TFU has no effect on recovery outcomes we cannot exclude an effect since this study was conducted in an ERAS setting. The women had already received thorough information about the perioperative care and postoperative symptoms and how to treat these before discharge. Thus, the standard TFU in this study would probably only confirm what the patient already knew and, therefore, would not add much to the recovery. On the contrary, the structured oriented coaching TFU was anticipated to have additional effects on the recovery since the coaching was intended to provide the patient with tools to better handle the perceived troublesome discomfort. Since all women received the same perioperative information according to the ERAS program and no differences were seen on any of the outcome measures between the controls and the three TFU groups, it is most likely that TFU and coaching as used in this study do not have an effect on postoperative symptoms and consumption of analgesics. Consequently, the external validity of the trial findings is restricted to settings using similar ERAS programs.

uTC and uV often reflects an unexpected deviation in the recovery for the patient [17].Women with a uTC followed by a visit to a health care provider reported higher pain intensity and a higher summary score of postoperative symptoms. Similar findings were seen in those with and without a following visit, whereas no differences were found between women with no uTC and those with a uTC without a following visit. The findings were independent of intervention, indicating that the interventions seemed to generate equal effects in these groups.

In spite of the TFU and the use of ERAS, the occurrence of uTCs and uVs was still rather high. This may indicate that having planned contacts even with weekly intervals does not reduce uTCs. We were not able to clearly establish the reasons for this phenomenon, but rapid onset of unforeseen symptoms between two TFU occasions may be a plausible explanation and is obviously not preventable by TFU. This might indicate an inability of the ERAS programs to counteract or prevent such unforeseen events. Although we do not know the specific reasons for the uTCs or the uVs, the results might indicate that pain and other symptoms are among the most common reasons for the contacts. Thus, our findings may indicate that the nurses giving telephone counseling in clinical practice were excellent and capable of discriminating alarming clinical symptoms from minor important symptoms that did not need a visit for clinical evaluation.

Patient satisfaction is gaining importance as a measure of quality of care but there is no consistent association between patient satisfaction and clinical outcomes after surgery [18]. Although TFU may increase patient satisfaction with post-discharge care [15], it seems debatable from a health-economic perspective to justify the consumption of resources for TFU without demonstrating clear benefits in clinical outcomes. We did not find such benefits in this study, and previously we have shown that TFU did not improve the health-related quality of life [9].

Postoperative recovery is multifactorial and efforts to improve it would probably benefit from being individualized, taking into account the patient’s psycho-social status and including prehabilitation. Prehabilitation, defined as the practice of enhancing a patient’s functional capacity before surgery, with the aim of improving postoperative outcomes [19–21], in association with TFU needs to be evaluated.

## Conclusion

The TFU strategy including the structured coaching program adhered to in this study did not seem to affect recovery from symptoms or consumption of analgesics after benign hysterectomy in an ERAS setting. uTCs and uVs were probably caused by increased pain or other symptoms but were not influenced or preventable by the TFU.

## Abbreviations

ANOVA: Analysis of variance
CBT: Cognitive behavior therapy
ERAS: Enhanced recovery after surgery
POSTHYSTREC: Post-hysterectomy recovery
RN: Research nurse
SPSQ: Swedish postoperative symptoms questionnaire
TFU: Telephone follow-up
uTC: Unplanned telephone contact
uV: Unplanned visit
